# Fluctuation Theorems for Heat Exchanges between Passive and Active Baths

**DOI:** 10.3390/e26060439

**Published:** 2024-05-23

**Authors:** Massimiliano Semeraro, Antonio Suma, Giuseppe Negro

**Affiliations:** Dipartimento Interateneo di Fisica, Università degli Studi di Bari and INFN, Sezione di Bari, Via Amendola 173, 70126 Bari, Italy; antonio.suma@uniba.it (A.S.); giuseppe.negro@ba.infn.it (G.N.)

**Keywords:** heat exchange, out-of-equilibrium systems, fluctuation theorem, active bath, out-of-equilibrium temperatures

## Abstract

In addition to providing general constraints on probability distributions, fluctuation theorems allow us to infer essential information on the role played by temperature in heat exchange phenomena. In this numerical study, we measure the temperature of an out-of-equilibrium active bath using a fluctuation theorem that relates the fluctuations in the heat exchanged between two baths to their temperatures. Our setup consists of a single particle moving between two wells of a quartic potential accommodating two different baths. The heat exchanged between the two baths is monitored according to two definitions: as the kinetic energy carried by the particle whenever it jumps from one well to the other and as the work performed by the particle on one of the two baths when immersed in it. First, we consider two equilibrium baths at two different temperatures and verify that a fluctuation theorem featuring the baths temperatures holds for both heat definitions. Then, we introduce an additional Gaussian coloured noise in one of the baths, so as to make it effectively an active (out-of-equilibrium) bath. We find that a fluctuation theorem is still satisfied with both heat definitions. Interestingly, in this case the temperature obtained through the fluctuation theorem for the active bath corresponds to the kinetic temperature when considering the first heat definition, while it is larger with the second one. We interpret these results by looking at the particle jump phenomenology.

## 1. Introduction

A fundamental open issue in statistical physics is the extension of the equilibrium framework to out-of-equilibrium settings. Amongst the many questions still waiting for an answer, the definition of a proper temperature that consistently regulates *heat fluctuations and exchanges* between out of equilibrium thermal baths posits a central problem. For glassy systems, whose non-equilibrium character is due to very long relaxational times [1,2], an effective thermal picture has already emerged. There, an effective temperature can in fact be defined using the non-equilibrium deviations of the fluctuation–dissipation theorem [3,4]. One then naturally wonders if a similar scenario also applies to other classes of out-of-equilibrium systems.

One class of out-of-equilibrium systems that, in the past few years, has attracted great interest is *active matter* [5,6,7,8,9,10,11,12,13,14,15,16,17]. The distinctive feature of all systems from this class is a continuous conversion and injection of energy from internal reservoirs or the surrounding environment into the system itself to produce self-propulsion of its minimal constituents. Interestingly, the mere introduction of a self-propulsion mechanism results in a wealth of new phenomena and features, for example, collective motion [13,18,19,20,21], motility-induced phase separation [22,23,24] a rich phase diagram [25,26,27,28] and dynamical phase transitions [29,30,31], most of which have no equivalent in passive counterparts. According to stochastic thermodynamics [32,33,34], the injection of energy is an irreversible process which makes active systems inherently out of equilibrium [35,36]. As a consequence, the Stokes–Einstein relation between injection and dissipation of energy is naturally violated at microscopic scales [37,38,39], therefore making active matter systems a perfect stage for introducing and testing different definitions of out-of-equilibrium temperatures. In this respect, we mention that there have been several attempts to describe this inherent non-equilibrium character at macroscopic scales through the introduction of an effective temperature [40,41,42,43,44,45,46]. However, up to our knowledge, a general effective thermal picture has not yet emerged.

An approach that can be exploited to test at the mesoscopic level different definitions of out-of-equilibrium temperatures is offered by the so called *fluctuation theorems*, i.e., universal constraints on the probability distribution of integrated observables like work, heat and entropy production evaluated along the trajectories of individual physical entities of the system of interest [32,47,48,49,50,51,52]. An important result showing that temperatures naturally enter heat fluctuation theorems is provided by [53], in which it is shown that the heat exchanged between two equilibrium thermal baths satisfies the following fluctuation theorem

(1) $$I\left(-q\right)-I\left(q\right)=\left(\frac{1}{T_{1}}-\frac{1}{T_{2}}\right)q\;,$$

where *q* is the heat exchanged per unit time, $I\left(q\right)\equiv \lim_{\tau ↑\infty }-\log P\left(q\right)/\tau$ is its associated rate function from Large Deviation Theory [54,55,56], and $T_{1}$,$T_{2}$ coincide with the bath temperatures. The above result was later studied in the context of Brownian particles [57,58], finding that its validity was in general restricted to finite intervals of *q*. The fluctuation theorem from Equation (1) represents a natural starting point for an investigation on the values $T_{1}$ and $T_{2}$ could take in (possibly still valid) fluctuation theorems in out-of-equilibrium contexts, where fluctuations are central. The temperatures defined from a fit of Equation (1) and denoted as $T_{FT}$ can in fact be compared with other significant definitions of temperature, such as the *effective temperature*, denoted as $T_{eff}$ and defined from the deviation of the fluctuation–dissipation theorem [3,4,40,41,45,59,60,61], or the *kinetic temperature*, instead denoted as $T_{kin}$ and defined from the equipartition theorem [4,41,46,62] (see Appendix A for more details).

**Figure 1:**
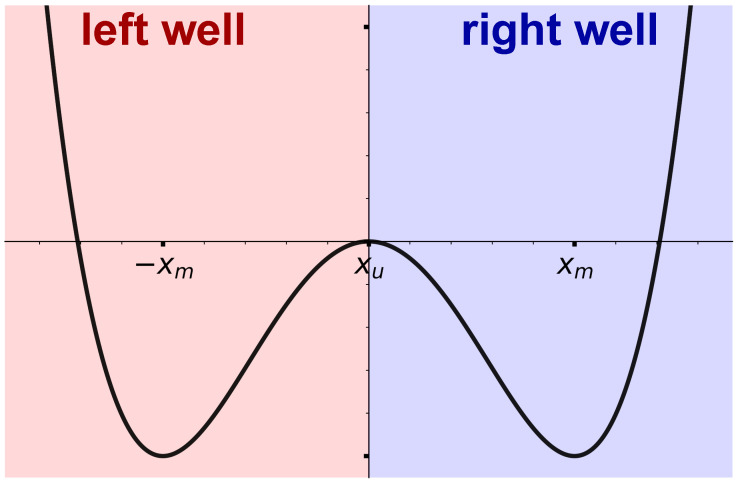
Schematic depiction of our idealized setup. The black line denotes the quartic double-well potential Equation (2) with minima and local maxima at $\pm x_{m}$ and $x_{u}$, respectively, and depth ΔU. The red and blue areas and labels below and above $x_{u}$ denote instead the action of baths with different features in the two wells.

Here, we numerically investigate the definition of $T_{FT}$ by considering an idealized setup which consists of a single one-dimensional particle moving in a quartic double-well potential (see Figure 1 for a schematic depiction). In each well, the particle is put in contact with a different overall thermal bath, thus experiencing a different temperature. In the right well, we place an equilibrium thermal bath which is formalized through a Gaussian zero-mean delta-correlated white noise plus a viscous friction force and satisfies a usual fluctuation–dissipation theorem with an effective temperature trivially coinciding with both the bath and the kinetic ones. For the left well bath, we instead consider two different cases: first, we fix a further equilibrium thermal bath with the same characteristics as the one in the right well except for a different temperature; then, we make it an active bath by fixing an equilibrium thermal bath analogous to the one from the right well, now with the same temperature, and introducing an Ornstein–Uhlenbeck process playing the role of an additional Gaussian coloured noise with exponential self-correlation. In this way, when in the left well, the particle effectively turns into an active particle, more specifically an active Ornstein–Uhlenbeck particle [63,64,65,66]. Moreover, we remark that in that case, neither the usual fluctuation–dissipation theorem is satisfied [38] nor kinetic and effective temperatures coincide [46]. The heat exchanged is measured according to two different definitions: as the work performed by one of the two baths on the particle and as the sum of the kinetic energies carried by the particle every time it jumps from one well to the other. The first definition is nothing but the usual heat as defined in the framework of stochastic thermodynamics [33,67]. The second one is instead newly introduced as suggested by our specific setup.

We find that in all cases considered the fluctuation theorem Equation (1) is still valid. In more detail, in the case in which two equilibrium thermal baths with different temperatures are fixed, both definitions of heat exchanged lead to the validity of Equation (1) with a slope in accordance with the bath temperatures. This first result provides an essential correspondence between $T_{FT}$ and both $T_{eff}$ and $T_{kin}$, which, as mentioned above, in this case both trivially coincide with the bath ones. In the active bath case, we find instead that different values of $T_{FT}$ associated with the active bath emerge based on the definition of heat under study. When the heat as a sum of kinetic energies is considered, the extracted $T_{FT}$ turns out to correspond to the kinetic temperature of the active bath. When instead considering the heat as the work performed by the thermal environment, this temperature assumes intermediate values between the kinetic temperature and the effective one. These results and discrepancies can be interpreted by looking at the particle jump phenomenology.

The remainder of the paper is structured as follows. In Section 2, we present the model and methods we adopted. In particular, in Section 2.1, we describe our setup and detail the two cases under scrutiny; in Section 2.2, we introduce the two definitions of heat exchanged we consider along with the energy balance of the system; in Section 2.3, we describe the numerical methods we adopted, and in Section 2.4, we comment on the stationary position distribution of the system. Next, in Section 3, we present and comment the results of our investigation for the two bath configurations considered. Finally, in Section 4, we report our closing remarks.

## 2. Model and Methods

### 2.1. Model

The general framework of our setup is that of a unidimensional unit-mass mesoscopic particle of position x(t) and diameter σ=1 moving under the action of the external quartic double-well potential

(2) $$U\left(x\left(t\right)\right)=\frac{a}{4}\;{\left(x\left(t\right)-x_{u}\right)}^{4}-\frac{b}{2}{\left(x\left(t\right)-x_{u}\right)}^{2}\;,$$

where a,b>0, and $x_{u}$ is the centre of the potential which we set to zero and which serves as a separating point between the regions $x>x_{u}$ (*right well*) and $x\leq x_{u}$ (*left well*), where thermal baths with different features act. The local maximum of the potential is located at $x_{u}=0$, the global minima are at $\pm x_{m}=x_{u}\pm \sqrt{b/a}$ distanced by $2\sqrt{b/a}$, and the potential depth $\Delta U=U\left(x_{u}\right)-U\left(\pm x_{m}\right)=b^{2}/4a$ represents the height of the barrier the particle has to overcome to hop from one well to the other. In order to highlight the spatial separation of the two baths induced by the potential Equation (2), we recast the usual Langevin equation describing the particle dynamics with initial conditions $x\left(0\right)\equiv x_{0}$ and $\dot{x}\left(0\right)\equiv v_{0}$ into the following form

(3) $$\ddot{x}\left(t\right)=B_{1}\left(\dot{x}\left(t\right),t\right)\theta \left(x\left(t\right)\right)+B_{2}\left(\dot{x}\left(t\right),t\right)\left(1-\theta \left(x\left(t\right)\right)\right)-\frac{dU[x(t)]}{dx}\;,$$

where $B_{1}\left(\dot{x}\left(t\right),t\right)$ and $B_{2}\left(\dot{x}\left(t\right),t\right)$ collect the forces exerted by the overall baths in the two wells. The presence of the Heaviside functions θ(x(t)) ensures in fact that $B_{1}\left(\dot{x}\left(t\right),t\right)$ and $B_{2}\left(\dot{x}\left(t\right),t\right)$ only act when the particle is in the right or left well, respectively. For the sake of simplicity, here, we assume the convention θ(0)=0 [68] instead of the half-maximum one θ(0)=1/2 [69], so that the function is left-continuous at x=0, and θ(t) and 1−θ(t) can be properly considered as the indicator functions of the intervals (0,+∞) and (−∞,0], respectively. We underline that this choice does not affect our results as the value of a function at a single point does not affect the overall values of the heat integrals from Section 2.2. Concerning instead the action of the baths, whenever the particle hops into each of the two wells, their corresponding noise processes are made to restart acting with an initial condition extracted from their stationary distributions. Figure 1 graphically summarizes our setup, highlighting with different colours the left and right well regions.

We now specify the actual composition of the forces contributing to each bath. In the right well, $B_{1}\left(\dot{x}\left(t\right),t\right)$ is always associated with a usual equilibrium thermal bath, hereafter referred to as *passive bath*, thus

(4) $$B_{1}\left(\dot{x}\left(t\right),t\right)=-\gamma \dot{x}\left(t\right)+\sqrt{2\gamma T_{1}}\;\xi_{1}\left(t\right)\;,$$

where γ is the viscous friction coefficient, $T_{1}$ is the bath temperature, and $\xi_{1}\left(t\right)$ is a usual Gaussian white noise with $\langle \xi_{1}\left(t\right)\rangle =0$ and $\langle \xi_{1}\left(t\right)\xi_{1}\left(t^{\prime }\right)\rangle =\delta (t-t^{\prime })$. Note that for the sake of simplicity, here and in the following, we set the Boltzmann constant $k_{B}$ to unity. The distribution for the restart of $\xi_{1}\left(t\right)$ is then a normal Gaussian N(0,1). As aforementioned, for the bath in the left well, we instead distinguish two different cases:

(a) another passive bath with friction coefficient γ and temperature $T_{2}$, i.e.,
  (5) $$B_{2}\left(\dot{x}\left(t\right),t\right)=-\gamma \dot{x}\left(t\right)+\sqrt{2\gamma T_{2}}\;\xi_{2}\left(t\right)\;,$$
  where $\xi_{2}\left(t\right)$ is a Gaussian white noise independent from $\xi_{1}\left(t\right)$ with $\langle \xi_{2}\left(t\right)\rangle =0$, $\langle \xi_{2}\left(t\right)\xi_{2}\left(t^{\prime }\right)\rangle =\delta (t-t^{\prime })$, and $T_{2}$ in general different from $T_{1}$. As for $\xi_{1}\left(t\right)$, the distribution for the restart of $\xi_{2}\left(t\right)$ is N(0,1). Note that in this specific case, the temperature for the entire domain can be written as the *x*-dependent function $T\left(x\right)\equiv T_{2}+\left(T_{1}-T_{2}\right)\theta \left(x\right)$, so that the overall Langevin equation Equation (3) can be recast as
  (6) $$\ddot{x}\left(t\right)=-\gamma \dot{x}\left(t\right)+\sqrt{2\gamma T[x(t)]}\;\xi \left(t\right)-\frac{dU[x(t)]}{dx(t)}\;,$$
  where ξ(t) is a single Gaussian white noise with 〈ξ(t)〉=0 and $\langle \xi \left(t\right)\xi \left(t^{\prime }\right)\rangle =\delta (t-t^{\prime })$ acting everywhere in the system which is made multiplicative by the presence of T(x) in its multiplicative factor;
(b) a passive bath with friction coefficient γ and temperature $T_{2}$ and an additional *Ornstein–Uhlenbeck noise* reminiscent of the active force from the active Ornstein–Uhlenbeck particle model [63,64,65,66] and hereafter referred to as *active bath*, i.e.,
  (7) $$B_{2}\left(\dot{x},t\right)=-\gamma \dot{x}\left(t\right)+\sqrt{2\gamma T_{2}}\;\xi_{2}\left(t\right)+a\left(t\right)\;,$$
  where $\xi_{2}\left(t\right)$ is a Gaussian white noise analogous to the one from case (a) and a(t) is an Ornstein–Uhlenbeck process implemented as the solution of the additional stochastic differential equation
  (8) $$\dot{a}\left(t\right)=-\gamma_{R}a\left(t\right)+F_{a}\sqrt{2\gamma_{R}}\;\eta \left(t\right)$$
  with initial condition $a\left(0\right)\equiv a_{0}$, where η(t) is a further Gaussian white noise independent from both $\xi_{1}\left(t\right)$ and $\xi_{2}\left(t\right)$ with 〈η(t)〉=0 and $\langle \eta \left(t\right)\eta \left(t^{\prime }\right)\rangle =\delta (t-t^{\prime })$, and $\gamma_{R}^{-1}$ and $F_{a}$ are the *persistence time* associated with the active process and a positive constant ruling its magnitude, respectively. From the average and self-correlation of a(t)
  (9) $$\langle a(t)\rangle =a_{0}e^{-\gamma_{R}t}\;\mathrm{and}\;\langle a\left(t\right)a\left(t^{\prime }\right)\rangle =a_{0}^{2}e^{-\gamma_{R}\left(t+t^{\prime }\right)}+F_{a}^{2}\left(e^{-\gamma_{R}\left|t-t^{\prime }\right|}-e^{-\gamma_{R}\left(t+t^{\prime }\right)}\right)\;,$$
  one in fact immediately realizes that $\tau_{p}=\gamma_{R}^{-1}$ controls the exponential decay of both average and self-correlations at large times and $\langle a^{2}\left(t\right)\rangle ≃F_{a}^{2}$, so that $F_{a}$ indeed plays the role of an average magnitude for the active process [64,66,70]. Equation (9) also suggests that the distributions for the restart of $\xi_{2}\left(t\right)$ and a(t) are N(0,1) and $N(0,F_{a}^{2})$, respectively. In order to better discern the action of a(t), here, we fix $T_{1}=T_{2}$ and for the sake of simplicity, we also set $\gamma_{R}=3T_{2}/\left(\gamma \sigma^{2}\right)$ [24,31,71]. Moreover, as typically done [23,24,25,31,62,71], we control the relative magnitude activity and thermal noise by varying the adimensional Péclet number
  (10) $$Pe\equiv \frac{F_{a}\sigma }{T_{2}}\;,$$
  where we recall σ=1 is the particle diameter. We remark that in general, the active bath configuration can be realized in actual experiments by using Janus particles [72,73,74,75] or optical tweezers [76,77], or by introducing a passive tracer particle in a suspension of active particles whose collisions with the tracer itself can be described by a(t) [52,78].

Finally, in order to allow the particle to correctly thermalize in each well before every jump, we need to correctly assess the relevant timescales of the system. Concerning case a), there are only two relevant timescales. The first one is the *inertial time* $\tau_{I}=\gamma^{-1}$, which is the typical time needed to attain thermal equilibrium with the bath. The second one is the average time the particle remains in one well before hopping starting the barrier ascension from $\pm x_{m}$, or *average residence time*, $\tau_{r}$. In the overdamped limit for a single white-noise bath acting everywhere and a parameter choice such that ΔU/T≫1, $\tau_{r}$ is estimated as [79]

(11) $$\tau_{r}=\frac{\pi \gamma }{\sqrt{U^{\prime \prime }\left(x_{m}\right)\left|U^{\prime \prime }\left(x_{0}\right)\right|}}e^{\frac{\Delta U}{T}}=\frac{\pi \gamma }{\sqrt{2}b}e^{\frac{\Delta U}{T}}\;,$$

where $U^{\prime \prime }\left(x\left(t\right)\right)$ is the second derivative of the potential Equation (2). In order to allow the particle to thermalize after each jump we require $\tau_{r}>\tau_{I}$ in each well. In the following, we use the symbols $\tau_{r}^{l}$ and $\tau_{r}^{r}$ to denote the average residence times in the left and right well, respectively. Concerning case (b), yet another timescale needs to be considered: the persistence time $\tau_{p}=\gamma_{R}^{-1}$ controlling the exponential decay of the coloured noise correlations. A further condition that is required to let the particle thermalise in the presence of the additional Ornstein–Uhlenbeck force is then $\tau_{r}>\tau_{p}$. We remark that in presence of an active process like a(t) a Kramers-like formula similar to Equation (11) for $\tau_{p}$ is still in place in some limiting conditions [80]. However, we checked in our settings that such a formula did not hold, thus forcing us to resort to numerical estimations.

### 2.2. Definitions of Heat Exchanged and Energy Balance

Our primary interest focuses on the heat exchanged between the two wells as the particle hops between them, which here we sample according to two different definitions capturing each different physical aspects of the system.

The first definition we consider relies on the intuitive idea that exchanges of energy and heat between the two baths must somehow be related to the jumps of the particle from one well to the other. More in detail, each of the $N_{E}\geq 0$ jumps occurring during a time interval of duration τ can be considered as an event of instantaneous transfer of kinetic energy from one bath to the other, with the particle playing the role of carrier. Therefore, an intuitive way in which we define the energy exchange between the two is

(12) $$Q_{E}^{R}\equiv \frac{1}{2}\sum_{j=1}^{N_{E}}|\dot{x}\left(\tau_{j}\right)|\dot{x}\left(\tau_{j}\right)\;.$$

We would like to stress that the above formula is simply configured as the sum of the kinetic energies carried during each jump by the particle, i.e., a simple quantitative version of the intuitive idea delineated above. Here, the subscript *E* denotes the energetic origin of this definition, while ${\left\{\tau_{j}\right\}}_{j=1,\ldots ,N_{E}}$ is the succession of times during the sampling interval of duration τ in which all jumps events occur, i.e., at which $x\left(t\right)=x_{u}$. Note that the absolute value in Equation (12) ensures the increments of $Q_{E}$ are given a proper sign depending on the direction of each jump event. For the right well, they are in fact positive (negative) when the particle jumps from left (right) to right (left), in agreement with the physical intuition that the right well bath receives (loses) energy when the particle enters in (goes away from) it. In order to remain faithful to the prescription that a bath acquires (loses) energy when the particle jumps in (away from) it, when focusing on the left well, we need to invert our point of view. In particular, now, the increments of $Q_{E}^{L}$ must be considered negative (positive) when the particle jumps from left (right) to right (left). In terms of the total energy exchange $Q_{E}^{L}$, this translates into an overall minus sign with respect to $Q_{E}^{R}$, i.e., $Q_{E}^{L}=-Q_{E}^{R}$. Trivially, $Q_{E}^{L}+Q_{E}^{R}=0$.

The second definition we consider takes up the usual one provided by stochastic thermodynamics in which heat is defined as the work performed on the particle by the passive bath, i.e., a viscous friction force plus white noise [33,67]. In our specific setting, the definition for the heat exchanged between particle and passive bath during a time interval of duration τ in the right well transforms into

(13) $$\begin{array}{cc} Q_{W}^{R} & \equiv -\int_{0}^{\tau }\;B_{1}\left(\dot{x}\left(s\right),s\right)\theta \left(x\left(s\right)\right)\;○\;dx\left(s\right)\\ \; & =-\sum_{j=1}^{N_{R}}\int_{\tau_{0,j}}^{\tau_{R,j}}\;B_{1}\left(\dot{x}\left(s\right),s\right)\dot{x}\left(s\right)\;ds\\ \; & =-\sum_{j=1}^{N_{R}}\int_{\tau_{0,j}}^{\tau_{R,j}}\;\left(-\gamma \dot{x}\left(s\right)+\sqrt{2\gamma T_{1}}\;\xi_{1}\left(s\right)\right)\dot{x}\left(s\right)\;ds \end{array}$$

where the symbol ○ denotes the adopted Stratonovich prescription [81], the minus sign denotes that it is the particle that performs work on the bath and ensures the same sign convention as for $Q_{E}^{R}$ is fulfilled, the subscript *W* highlights the thermodynamical origin of this definition, $N_{R}$ denotes the number of times the particle resides in the right well and $\tau_{0,j},\tau_{R,j},\;j=1,\ldots ,N_{R}$ denote the beginning and ending times of the *j*th residency in the well, respectively, with $\tau_{R,j}-\tau_{0,j}>0$ its duration.

**Figure 2:**
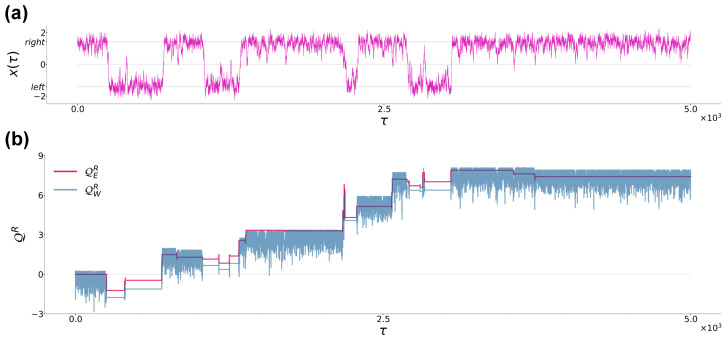
(**a**): Typical trajectory of a Brownian particle from case (a) at sampling time $\tau =5\times 10^{3}$. The black dashed lines denote the location of the left and right potential minima at $\pm x_{m}=\pm \sqrt{b/a}=\pm \sqrt{2}$. (**b**): Time evolution of $Q_{E}^{R}$ and $Q_{W}^{R}$ corresponding to the trajectory in panel (**a**). Parameters are a=1.0, b=2.0, γ=10, $T_{1}=0.2$ and $T_{2}=0.3$.

In order to provide some physical intuition about the difference between the heat definitions from Equations (12) and (13), in Figure 2a we show a typical particle trajectory in case (a), while in Figure 2b we show the corresponding realisations of $Q_{E}^{R}$ and $Q_{W}^{R}$ during the same time interval (numerical data are obtained using the numerical techniques described in Section 2.3). Note that $Q_{E}^{R}$ is piecewise continuous and presents discontinuous variations only when jump events occur, while $Q_{W}^{R}$ continuously evolves when the particle is in the right well, remaining constant when the particle jumps in the left well, and showing significant variations only when jump events occur. Note also that during the first permanence of the particle in the left well, $Q_{W}^{R}$ averages to zero, in agreement with the fact that during that time interval, the particle is thermalized with the right-well bath and the latter has not yet received any energy injection from the left-well bath.

Following standard procedures, from Equation (13) the trajectory-wise energy balance of the system can be obtained [33,67]. By simply using the Langevin equation Equation (3) to replace $B_{1}\left(\dot{x}\left(t\right),t\right)\theta \left(x\left(t\right)\right)$ in Equation (13) for generality in case (b) and adopting the Stratonovich prescription to calculate integrals [81], one in fact finds

(14) $$\begin{array}{cc} \frac{1}{2}\Delta {\dot{x}}^{2}\left(\tau \right)+\Delta U\left(x\left(\tau \right)\right) & =\int_{0}^{\tau }B_{1}\left(\dot{x}\left(s\right),s\right)\theta \left(x\left(s\right)\right)\dot{x}\left(s\right)\;ds+\int_{0}^{\tau }B_{2}\left(\dot{x}\left(s\right),s\right)(1-\theta \left(x\left(s\right)\right)\dot{x}\left(s\right)\;ds\\ \; & =-Q_{W}^{R}-Q_{W}^{L}+W_{a}\;, \end{array}$$

where $\int_{0}^{\tau }\;\ddot{x}\left(s\right)\dot{x}\left(s\right)\;ds=\left({\dot{x}}^{2}\left(\tau \right)-{\dot{x}}^{2}\left(0\right)\right)/2\equiv \frac{1}{2}\Delta {\dot{x}}^{2}\left(\tau \right)$ and $\int_{0}^{\tau }\;\frac{dU(x(s))}{dx(s)}\dot{x}\left(s\right)\;ds=\left(U\left(x\left(\tau \right)\right)-U\left(x\left(\tau \right)\right)\right)\equiv \Delta U\left(x\left(\tau \right)\right)$, respectively, denote the variation in kinetic and potential energy from the initial configuration at s=0 and final one at s=τ, $Q_{R}^{W}$ denotes the work performed by the particle on the right passive bath defined in Equation (13),

(15) $$\begin{array}{cc} Q_{W}^{L} & \equiv -\int_{0}^{\tau }\;\left(-\gamma \dot{x}\left(s\right)+\sqrt{2\gamma T_{2}}\;\xi_{2}\left(s\right)\right)\left(1-\theta \left(x\left(t\right)\right)\right)\;○\;dx\left(s\right)\\ \; & =-\sum_{j=1}^{N_{L}}\int_{\tau_{0,j}}^{\tau_{L,j}}\;\left(-\gamma \dot{x}\left(s\right)+\sqrt{2\gamma T_{2}}\;\xi_{2}\left(s\right)\right)\dot{x}\left(s\right)\;ds \end{array}$$

denotes the work performed by the particle on the passive component of the left bath only, i.e., the friction force plus white noise, with $N_{L}$ the number of times the particle resides in the left well, and $\tau_{0,j}<\tau_{L,j}$ the beginning and ending times of each of the *j*th residencies, and finally

(16) $$W_{a}\equiv \int_{0}^{\tau }\;a\left(s\right)\left(1-\theta \left(x\left(s\right)\right)\right)\;○\;dx\left(s\right)=\sum_{j=1}^{N_{L}}\int_{\tau_{0,j}}^{\tau_{L,j}}\;\;a\left(s\right)\dot{x}\left(s\right)\;ds$$

denotes the active work, i.e., the work performed by the additional noise in the left well providing a measure of the energy cost to sustain the particle self-propulsion [31,52,66,82,83]. In both Equations (15) and (16), ○ again underlies the Stratonovich prescription. We point out that $Q_{W}^{R,L}$ and $W_{a}$ are energy contributions extensive in time, while the variation in both kinetic and potential energies $\Delta {\dot{x}}^{2}\left(\tau \right)/2$ and ΔU(x(τ)) are not, i.e.,

(17) $$\lim_{\tau ↑\infty }\frac{1}{\tau }\int_{0}^{\tau }d\left(\frac{1}{2}{\dot{x}}^{2}\left(t\right)+U\left(x\left(t\right)\right)\right)=0\;,$$

or, by assuming ergodicity,

(18) $$\frac{d}{dt}\langle \frac{1}{2}{\dot{x}}^{2}\left(t\right)+U\left(x\left(t\right)\right)\rangle =0\;,$$

where the derivative is zero due to $\langle {\dot{x}}^{2}\left(t\right)/2+U\left(x\left(t\right)\right)\rangle$ assuming a constant value independent of *t*. As a consequence, when passing to the energy balance per unit time, at times much larger than all relevant timescales of the system, one has

(19) $$0=-q_{W}^{R}-q_{W}^{L}+w_{a}\;.$$

where q=Q/τ for all sub- and superscripts, and $w=W_{a}/\tau$. Note that, coherent with the fact that the system under consideration is globally isolated, Equation (19) shows the overall energies exchanged by the two baths, $-q_{W}^{R}$ for the right and $-q_{W}^{L}+w_{a}$ for the left one, to be of opposite signs and to sum to zero.

Finally, a few comments and remarks. We underline that case (a) does not include the additional noise a(t), so that $W_{a}=0$, and Equation (14) reduces to the usual equivalence between the energy variation of the system and the heat exchanged. We would also like to stress that in case (b), $Q_{W}^{L}$ does not capture the heat exchanges related to the left bath in its entirety. As $-\int_{0}^{\tau }B_{2}\left(\dot{x}\left(s\right),s\right)\dot{x}\left(s\right)\left(1-\theta \left(x\left(s\right)\right)\right)\;ds=Q_{W}^{L}-W_{a}$, the latter in fact also includes the active work contribution. Nevertheless, as shown in Section 3.2, $Q_{W}^{L}$ is indirectly influenced by the action of the active noise as the latter clearly affects the particle velocity in the left well. In this respect, we remark that as the active noise a(t) pushes the particle, it is very likely for a(t) and $\dot{x}\left(t\right)$ to have the same sign so that $W_{a}$ from Equation (16) is very unlikely to be negative. Finally, referring to the trajectory of $Q_{W}^{R}$ relative to case (a) from Figure 2b, we conclude by pointing out that during each permanence of the particle in the right well, $Q_{W}^{R}$ is bounded from above by a different value. In order to prove this point, let us consider a particle which has jumped into the right well the last time at $\tau_{J}$ and up to time $\tau >\tau_{J}$, remained in it. Then, one has

(20) $$\begin{array}{cc} Q_{W}^{R} & =c_{q}-\int_{\tau_{J}}^{\tau }\;\left(-\gamma \dot{x}\left(s\right)+\sqrt{2\gamma T_{1}}\;\xi_{1}\left(s\right)\right)\dot{x}\left(s\right)\;ds\\ \; & =\left(\frac{1}{2}{\dot{x}}^{2}\left(\tau_{J}\right)+U\left(x\left(\tau_{J}\right)\right)\right)-\left(\frac{1}{2}{\dot{x}}^{2}\left(\tau \right)+U\left(x\left(\tau \right)\right)\right)+c_{q}\\ \; & \leq \frac{1}{2}{\dot{x}}^{2}\left(\tau_{J}\right)+U\left(x\left(\tau_{J}\right)\right)-U_{m}+c_{q}\;, \end{array}$$

where in the first row we used the definition Equation (13) and $c_{q}$ records the value accumulated by $Q_{W}^{R}$ up to time $\tau_{J}$, in the second row we instead used the Langevin equation Equation (3) and performed an integration similarly to the case of Equation (14), and finally, in the third row we used the lower bounds ${\dot{x}}^{2}\left(\tau \right)/2\geq 0$ and $U\left(x\left(\tau \right)\right)\geq U\left(\pm x_{m}\right)\equiv U_{m}$. Interestingly, the bound of $Q_{W}^{R}$ from Equation (20) comes to depend on $c_{q}$, which englobes the integration of $Q_{W}^{R}$ up to time $\tau_{J}$, on the potential energy $U_{m}$ at the minimum and also on the kinetic and potential energies evaluated exactly at $\tau_{J}$, i.e., when the particle last entered into the right well.

### 2.3. Numerical Methods and Parameters

The numerical integration of Equations (3) and (8) was performed via the velocity Verlet [84] and the Euler–Maruyama [85] integrators, respectively, with integration timestep $dt=10^{-2}$ in both cases. We chose for the quartic potential Equation (2) a=1 and b=2, then setting a distance between minima and a barrier height of $2\sqrt{2}$ and ΔU=1, respectively. Along with the unitary particle mass and diameter σ, the barrier height ΔU set the reduced units of our simulations. In all cases, we fixed γ=10 and $T_{1}=0.2$, while in case (b), we fixed $\gamma_{R}=3T_{2}/\left(\gamma \sigma^{2}\right)$ with $T_{2}=T_{1}=0.2$ and varied Pe by acting on $F_{a}$. The inertial, persistence, and right residence times therefore resulted $\tau_{I}=0.1$, $\tau_{p}∼16.67$ and $\tau_{r}^{r}=1.65\times 10^{3}$. The specific choices for $T_{2}$ in case (a) and $F_{a}$ in case (b) and consequent left-well residence times are instead specified case by case. We evolved the system for time intervals of duration τ, in the following referred to as sampling time, up to $\tau =3\times 10^{4}$, the latter in all cases considered much larger than all of the characteristic times of the system.

We sampled the heat per unit time q=Q/τ for different sampling times τ according to the two definitions Equations (12) and (13) (as in the definitions from Section 2.2, in the following, the subscripts E,W and superscripts L,R will specify on a case-by-case basis which heat in which well is being considered). The heat distributions p(q) were obtained by considering $N_{p}=10^{6}$ independent trajectories previously evolved for a time $\tau_{eq}=10^{4}$ much larger than all of the characteristic time so as to always start from the stationary configuration. Taking into account that whenever *q* satisfies a large-deviation principle its distributions takes the asymptotic form $p\left(q\right)≍e^{-\tau I(q)}$, with ≍ the asymptotic equivalence symbol underlying sub-exponential contributions c(q) and I(q) rate function [54,55,56], these distributions were then used to check the validity of the fluctuation theorem Equation (1) by evaluating the ratio

(21) $$\begin{array}{cc} \frac{1}{\tau }\log\left(\frac{p(q)}{p(-q)}\right) & ≍I\left(-q\right)-I\left(q\right)=\left(\frac{1}{T_{r}}-\frac{1}{T_{l}}\right)q\;, \end{array}$$

where I(−q)−I(q) is the rate function difference appearing in Equation (1), and $T_{r}$ and $T_{l}$ denote the temperatures associated with the right and left well, respectively. Note that the symbol ≍ underlies at finite times the appearance of the ratio (c(q)−c(−q))/τ, which becomes increasingly negligible as time flows. Operatively, the estimates for $T_{r}$ and $T_{l}$, in the following denoted as $T_{FT}$, were obtained by first evaluating the ratio in the left-hand side of Equation (21) with our numerical distributions p(q) at different τ’s and then performing at each of these times a linear fit of the resulting curves. Without loss of generality, in the following, we consider settings in which the slope in Equation (21) is positive, corresponding to $T_{l}>T_{r}$. While for case (a) one intuitively expects $T_{r}$ ($T_{l}$) to coincide with $T_{1}$ ($T_{2}$) (a circumstance which is indeed verified in Section 3.1), for case (b), we have no a priori indications for the values they could take in the presence of the active bath, especially for $T_{l}$. In order to extract a $T_{FT}$ estimate for the left well, motivated by the results for case (a), we assumed $T_{r}=T_{1}$, extracted $T_{FT}=T_{l}$ from a fit of Equation (21) and compared it with the effective and kinetic temperatures $T_{eff}$ and $T_{kin}$, in turn numerically sampled according to their definitions from Appendix A.

To conclude, we remark that sampling both positive and negative values of heat becomes increasingly more difficult as the difference between the relevant temperatures of the two baths is made larger. Therefore, in the following, we implemented parameter choices for which such a sampling is numerically feasible.

### 2.4. Stationary Position Distribution

Before presenting our results, let us comment about the stationary position distribution in the two cases under consideration. These distributions, which we recall can be obtained as the solution of the Fokker–Planck equation with time derivative set to zero [86], provide in fact useful insights on average residence times, in turn useful for our later discussion.

**Figure 3:**
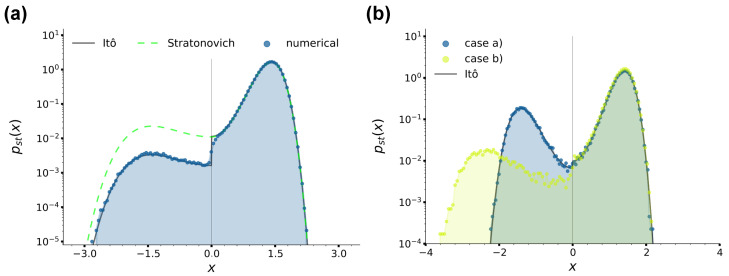
(**a**): Stationary position distributions for case (a), with $T_{1}=0.2$ and $T_{2}=1.4$ at sampling time $\tau =3\times 10^{4}$. The black solid and green dashed lines are the stationary solutions Equation (22) and Equation (23), respectively, while the blue histogram is the position distribution numerically sampled, as denoted by the legend. (**b**): Stationary position distributions for cases (a) and (b) and Equation (22), as denoted by the legend. For case (a), we fix $T_{1}=0.2,\;T_{2}=0.3$, while for case (b), $T_{1}=T_{2}=0.2,\;Pe=50$. In all panels, we fixed γ=10 and a=1.0, b=2.0.

Concerning case (a), in the overdamped limit and under the Itô prescription, the drift and diffusion coefficients of the Fokker–Planck equation are $-\gamma^{-1}U^{\prime }\left(x\right)$ and $\gamma^{-1}T\left(x\right)$ [86,87], respectively, with T(x) the x-dependent temperature defined in Section 2.1. The resulting stationary Fokker–Planck equation has the following solution

(22) $$p_{st}\left(x\right)=\frac{N_{I}}{T(x)}e^{-\frac{U(x)}{T(x)}}\;,$$

with $N_{I}$ a normalisation factor and U(x) the quartic potential Equation (2), which is clearly reminiscent of the equilibrium Boltzmann distribution. This solution is obtained by first replacing T(x) with a continuous parameter-dependent function $T_{\epsilon }\left(x\right)$ such that $T\left(x\right)=\lim_{\epsilon ↓0}T_{\epsilon }\left(x\right)$, then following the standard procedure for the solution of the stationary Fokker–Planck equation with $T_{\epsilon }\left(x\right)$, and finally taking the limit ϵ↓0. Note that $p_{st}\left(x\right)$ shows a jump discontinuity at $x_{u}$ when $T_{1}\neq T_{2}$, which disappears when $T_{1}=T_{2}$, i.e., in the usual case of a Brownian particle under the effect of just one equilibrium thermal bath. The associated discontinuity height is $\Delta p_{st}=|\lim_{x↑x_{u}}p_{st}\left(x\right)-\lim_{x↓x_{u}}p_{st}\left(x\right)|=N_{I}|T_{2}^{-1}-T_{1}^{-1}|$ and becomes more and more marked as the difference $|T_{1}-T_{2}|$ is increased. Note also that the two temperatures $T_{1}$ and $T_{2}$ determine the shape and height of the distribution in each well, but they play no role in the maxima locations, which in turn come to coincide with the potential minima at $\pm x_{m}=x_{u}\pm \sqrt{b/a}$. For the sake of completeness, we mention that under the Stratonovich prescription, the diffusion coefficient of the Fokker–Planck equation remains unaltered, while its drift coefficient becomes $\gamma^{-1}\left(-U^{\prime }\left(x\right)+T\left(x\right)\right)$, so that the stationary solution is now

(23) $$p_{st}\left(x\right)=N_{S}e^{-\frac{U(x)}{T(x)}}\;,$$

with $N_{S}$ a normalisation factor, which, contrary to Equation (22), is always continuous at $x_{u}$ also when $T_{1}\neq T_{2}$. We would like to stress that the difference between Equations (22) and (23) can be ultimately traced back to the presence of two regions with different temperatures. In fact, as mentioned in Section 2.1, in case (a), the Langevin equation Equation (3) can be recast as Equation (6), which is characterised by a multiplicative noise due to the presence of the *x*-dependent temperature T(x). Therefore, as well known from the literature [86,88], applying different integration schemes leads to different results, hence the different drift coefficients for the stationary Fokker–Planck equation in the Itô and Stratonovich prescriptions and the resulting different stationary distributions Equations (22) and (23). In Figure 3a, we provide a comparison between these two stationary solutions and the numerical position distributions at $\tau =3\times 10^{4}$ obtained by integrating the equations of motions as described in Section 2.3 and setting $T_{1}=0.2$ and $T_{2}=1.4$, so that $|T_{1}-T_{2}|∼1$. The figure at the same time shows that the numerical algorithms we used perform the integration under the Itô prescription and confirms the presence of the jump discontinuity in Equation (22).

In Figure 3b, we report instead a comparison between the numerical stationary position distributions for cases (a) and (b). For case (a) we choose $T_{1}=0.2$, $T_{2}=0.3$, while for case (b), we fix $T_{1}=T_{2}=0.2$ and $F_{a}=10$ (Pe=50) so that, as is shown in Section 3.2, the kinetic temperature in the left well is ∼$0.3=T_{2}$ (the reason for considering the kinetic temperature will appear clear in Section 3.2). Note that in the left well for case (b), the location $-{\tilde{x}}_{m}$ of the peak of the distribution is shifted towards the left with respect to the location of the potential minimum $-x_{m}$ due to the persistent pushing of the active noise. Even though, up to our knowledge, the stationary Fokker–Planck equation in case (b) has no exact analytical solution, our numerical results are coherent with the ones from [70], in which a single active Ornstein–Uhlenbeck particle in a quartic double-well potential like Equation (2) is studied. In particular, in [70] the location of the peaks of the distribution are identified as the points in which the confining force due to the quartic potential Equation (2) and the active force approximated by its average magnitude $F_{a}$ are balanced, i.e., as the solutions of the equation $-ax^{3}+bx=\pm F_{a}$, where the ± signs apply to the right and left wells, respectively. In our case, the solution of the above equation relative to the left well gives $-{\tilde{x}}_{m}≃2.46$, which is in good agreement with the location of the left peak from Figure 3b.

To conclude, we point out that the distributions we just commented on provide qualitative insights on the average residence time $\tau_{r}^{r}$ and $\tau_{r}^{l}$ of the particle in each well, which are essential information especially for case (b) in which an analytic estimate for $\tau_{r}^{l}$ is not available. It is in fact intuitive to see that in general, apart from the specific distribution features, lower temperatures associated with higher peaks in the distributions imply larger residence times, and vice versa for higher temperatures. According to Figure 3b, we then intuitively expect that the average residence times in the cases under consideration rank as follows: $\tau_{r}^{r}$ is the largest, $\tau_{r}^{l}$ in case (a) is intermediate and finally, $\tau_{r}^{l}$ in case (b) is the shortest.

## 3. Results

### 3.1. Heat Exchanges between Two Passive Baths

**Figure 4:**
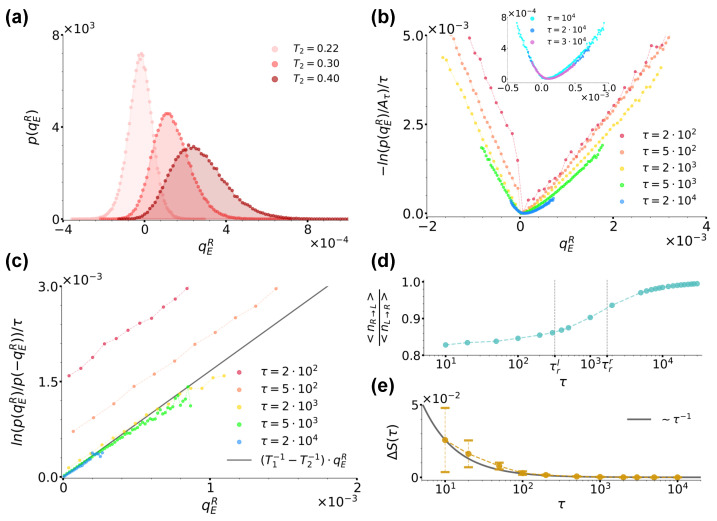
(**a**): Distribution $p(q_{E}^{R})$ for case (a) at sampling time $\tau =3\times 10^{4}$ for $T_{2}=0.22,\;0.3$ and 0.4, as denoted by the legend. (**b**): Curves $-\ln(p\left(q_{E}^{R}\right)/A_{\tau })/\tau$ for $T_{2}=0.3$ at different sampling times, as denoted by the legend. $A_{\tau }$ denotes the maximum of the distribution at each sampling time. The inset shows instead the trend of the same curves at the largest sampling times considered. (**c**): Ratio $\ln(p\left(q_{E}^{R}\right)/p\left(-q_{E}^{R}\right))/\tau$ evaluated at different sampling times τ using data from panel (**b**) along with the right hand-side of Equation (21) plotted with $T_{r}=T_{1}=0.2$ and $T_{l}=T_{2}=0.3$, as denoted by the legend. (**d**): Ratio between the average number of jumps in the right→left direction and left→right directions denoted by $\langle n_{R\to L}\rangle$ and $\langle n_{L\to R}\rangle$, respectively, as functions of sampling time. The dashed lines denote the left and right average residence times $\tau_{r}^{l}=3.11\times 10^{2}$ and $\tau_{r}^{r}=1.64\times 10^{3}$, respectively. (**e**): Shift ΔS(τ) of the curves from panel (**c**) as a function of time. For comparison, here, the black solid line reports the trend of $∼\tau^{-1}$. In all panels, we fixed γ=10, $T_{1}=0.2$ and a=1.0,b=2.0.

We start in this section with the investigation of case (a) envisaging a passive bath in each of the two wells. We first fixed $T_{1}=0.2$ and considered three $T_{2}$ values, 0.22, 0.3 and 0.4. According to Equation (11), the corresponding average residence times $\tau_{r}^{l}$ are much larger than the inertial time $t_{I}=0.1$, ranging from $\tau_{r}^{l}=1.05\times 10^{3}$ for $T_{2}=0.22$ to $\tau_{r}^{l}=1.35\times 10^{2}$ for $T_{2}=0.4$. In Figure 4a, we show the distribution $p(q_{E}^{R})$ for these three choices of temperatures at sampling time $\tau =3\times 10^{4}$ (the distributions $p(q_{E}^{L})$ are just symmetrical). Note that all distributions are characterised by a positive average value, ∼$2.10\times 10^{-5}$ for $T_{2}=0.22$, ∼$1.34\times 10^{-4}$ for $T_{2}=0.3$ and ∼$2.68\times 10^{-4}$ for $T_{2}=0.4$, confirming that, as intuitively expected, on average, the colder bath in the right well receives more energy from the hotter bath in the left well than the one it outputs towards it through the jumping particle. Note also that the distributions are characterised by an increasing skewness as the temperature difference $\Delta T=|T_{1}-T_{2}|$ is increased. In the remainder of the present section, we focus on the case $T_{1}=0.2,\;T_{2}=0.3$, which at the same time guarantees an appreciable skewness of $p(q_{E}^{R})$ as well as an efficient sampling of both positive and negative heat values. Up to what our simulations afforded us to sample, we checked that the following results for this case also applied to the other values of $T_{2}$.

In order to study the validity of Equation (1), we first focused on the trend of $-\ln(p\left(q_{E}^{R}\right))/\tau$, which in the large time limit converges to the rate function $I(q_{E}^{R})$ whenever $q_{E}^{R}$ satisfies a large-deviation principle [54,55,56]. More specifically, in Figure 4b, we report $-\ln(p\left(q_{E}^{R}\right)/A_{\tau })/\tau$ extracted at different sampling times τ’s, as reported by the legend. At each sampling time, $A_{\tau }$ denotes the maximum of the distribution and in the ratio, it makes the resulting curves shift vertically so as to have a minimum value of zero. As highlighted by the inset of Figure 4b, for $\tau >10^{4}\gg \tau_{I}=0.1$, we observe that the curves do overlap, thus implying that $q_{E}^{R}$ satisfies a large-deviation principle. We therefore proceeded to check the validity of Equation (1) as prescribed by Equation (21). We used data from Figure 4b and report the resulting curves at the same τ’s in Figure 4c, as denoted by the legend. We found that at all τ’s, these curves were linear and, within numerical error, with a slope in agreement with $1/T_{1}-1/T_{2}$, so that we could identify $T_{r}$ with $T_{1}$ and $T_{l}$ with $T_{2}$. We would like to underline that while previous results proved a fluctuation theorem like Equation (1) to stand in the case of two different thermal baths separately at equilibrium but acting simultaneously everywhere in the system [53,57,58], our results extend this scenario to the case of spatially separated baths.

Interestingly, Figure 4c also shows that at short times, the numerical lines in Figure 4c do not cross the origin, but rather present a time-decreasing positive shift ΔS(τ) which we can explain by looking at the system phenomenology at short times. During the evolution of our system, three timescales come into play, i.e., the left and right well residence times, $\tau_{r}^{l}≃3.11\times 10^{2}<\tau_{r}^{r}≃1.64\times 10^{3}$, and the sampling time τ at which the distribution $p(q_{E}^{R})$ is considered. When taking into account a large number $N_{p}$ independent realizations of the system, one then intuitively expects that, as long as $\tau <\tau_{r}^{l}$, more jumps from left to right occur than in the opposite direction, while when $\tau_{r}^{l}<\tau <\tau_{r}^{r}$, the number of right→left jumps starts increasing until essentially matching the number of left→right ones at $\tau \gg \tau_{r}^{l},\tau_{r}^{r}$. Figure 4d confirms this intuition by showing the trend of the numerical ratio between the average number of jumps in the right→left and in the left→right directions, respectively denoted as $\langle n_{R\to L}\rangle$ and $\langle n_{L\to R}\rangle$, as functions of sampling time τ. The curve in fact starts from a value lower than one for $\tau <\tau_{r}^{l}$, which then it reaches asymptotically from below when $\tau \gg \tau_{r}^{r}$. This jump phenomenology clearly bears consequences on the distribution $p(q_{E}^{R})$, and then on the resulting fluctuation theorem. In fact, as apparent from Figure 4b, at short times $\tau <\tau_{r}^{l}$, its left and right branches weigh differently positive and negative heat values, the left branch being further away from its large-time stationary form than the right one and mirroring the jump imbalance biased towards left→right positive heat jumps. As mentioned in Section 2.3, when commenting about Equation (21), these effects are encoded in the distribution as a sub-exponential contribution $c(q_{E}^{R})$, which is a function of $q_{E}^{R}$ scaling as $t^{\alpha }$ with α<1 and in our case, which is directly related with the observed shift ΔS(τ). As shown in Figure 4e, we in fact find $\Delta S\left(\tau \right)=(c\left(q_{E}^{R}\right)-c\left(-q_{E}^{R}\right))/\tau$ decreasing as $≃\tau^{-1}$ corresponding to α≃0, the latter value signalling that the difference $c\left(q_{E}^{R}\right)-c\left(-q_{E}^{R}\right)$ is of order ∼O(1).

**Figure 5:**
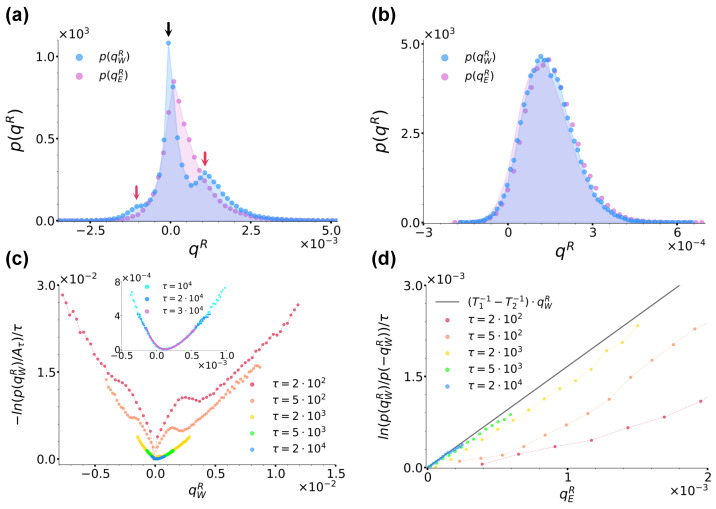
(**a**,**b**): Comparison between the distributions $p(q_{E}^{R})$ and $p(q_{W}^{R})$ at sampling times $\tau =10^{3}$ and $\tau =3\times 10^{4}$, respectively, with the same parameters as in Figure 4. In panel (**a**), the three arrows highlight the three peaks of $p(q_{W}^{R})$. (**c**): Curves $-\ln(p\left(q_{W}^{R}\right)/A_{\tau })/\tau$ for $T_{2}=0.3$ at different sampling times, as denoted by the legend. As in Figure 4, $A_{\tau }$ denotes the maximum of the distribution at each sampling time. The inset shows instead the trend of the same curves at the largest sampling times considered. (**d**): Ratio $\ln(p\left(q_{W}^{R}\right)/p\left(-q_{W}^{R}\right))/\tau$ evaluated at different sampling times using data from panel (**c**) along with the right hand-side of Equation (21) plotted with $T_{r}=T_{1}=0.2$ and $T_{l}=T_{2}=0.3$, as denoted by the legend. In all panels, we fixed γ=10, $T_{1}=0.2$ and a=1.0,b=2.0.

We now discuss the validity of the fluctuation theorem from Equation (1) for $q_{W}^{R}$ by studying $p(q_{W}^{R})$ in comparison with $p(q_{E}^{R})$. In Figure 5a,b, we compare the distributions $p(q_{W}^{R})$ and $p(q_{E}^{R})$ for the same parameter choice as in Figure 4b at sampling times $\tau =10^{3}$ and $\tau =3\times 10^{4}$, respectively. Let us focus on Figure 5a first. What immediately catches the eye is that, contrary to $p(q_{E}^{R})$ and as highlighted by the vertical arrows, $p(q_{W}^{R})$ is characterised by three peaks. This peculiar structure can be readily explained by recalling the jump phenomenology discussed above. The left and right external peaks highlighted by the red arrows are due to particles leaving and entering the right well, which are then responsible for negative and positive energy exchanges, respectively. Since here, $\tau_{r}^{l}<\tau <\tau_{r}^{r}$, more particles have jumped from left to right than in the opposite direction, hence the higher right peak. However, in our large sample of $N_{p}$ independent realizations, at that time, a large number of particles have not yet jumped at all from the right well, but rather have been exchanging an average zero heat with the equilibrium thermal bath in that well, hence the central peak located at $q_{W}^{R}=0$ highlighted by the black arrow. At large times, $p(q_{W}^{R})$ instead loses its three-peak structure and comes to coincide with $p(q_{E}^{R})$ from Figure 4b. In particular, the central peak disappears because at large times, it is extremely probable that all particles have already jumped almost once, while the other two become closer and closer until eventually merging. This overall scenario is graphically confirmed and clarified by Figure 5c, which reports the curves $-\ln(p\left(q_{W}^{R}\right)/A_{\tau })/\tau$ extracted at different sampling times τ’s, as denoted by the legend. The figure, in fact, at the same time shows the two external peaks clearly getting closer until eventually merging and also the curves converging towards a convex rate function $I(q_{W}^{R})$. Combining further this last information with the content of Figure 4b and Figure 5b, we can therefore affirm that at large times, $I\left(q_{W}^{R}\right)=I\left(q_{E}^{R}\right)$. The curves from Figure 5c allow us to finally check the validity of a fluctuation theorem for $q_{W}^{R}$ as prescribed by Equation (21). Figure 5d reports the ratio $\ln(p\left(q_{W}^{R}\right)/p\left(-q_{W}^{R}\right))/\tau$ evaluated at different sampling times using data from Figure 5c and, as highlighted by the black line reporting Equation (21) plotted with $T_{r}=T_{1}=0.2$ and $T_{l}=T_{2}=0.3$, shows that at large sampling time τ, $q_{W}^{R}$ indeed satisfies the same fluctuation theorem shown in Figure 4c and satisfied by $q_{E}^{R}$ with the same slope. Note that at short times, the fluctuation theorem is not satisfied because of the sub-exponential contribution $(c\left(q_{W}^{R}\right)-c\left(-q_{W}^{R}\right))/\tau$, which encodes the three-peak structure of $p(q_{W}^{R})$ and makes the curve actually curvilinear rather than rigidly vertically shifted. To conclude, we report that for the left well, we checked that $p\left(q_{W}^{L}\right)=p\left(-q_{W}^{R}\right)$ and consequently, that the same results discussed until this point for $q_{W}^{R}$ symmetrically still applied, so that the energy balance Equation (19)’s results were satisfied.

### 3.2. Heat Exchanges between a Passive and an Active Bath

**Figure 6:**
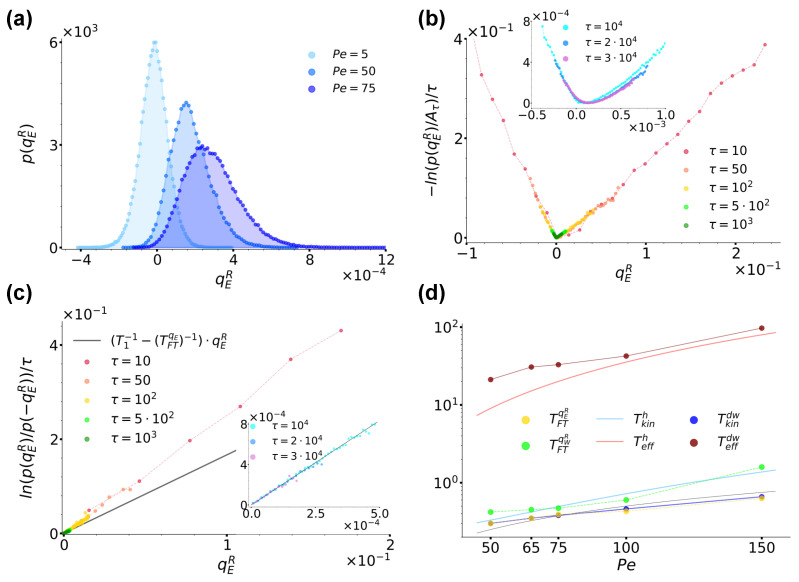
(**a**): Distribution $p(q_{E}^{R})$ for case (b) at sampling time $\tau =3\times 10^{4}$ for Pe=5,50 and 75, as denoted by the legend. (**b**): Curves $-\ln(p\left(q_{E}^{R}\right)/A_{\tau })/\tau$ for Pe=50 at different sampling times, as denoted by the legend. As in Figure 4, $A_{\tau }$ still denotes the maximum of the distribution at each sampling time. The inset shows instead the trend of the same curves at the largest sampling times considered. (**c**): Ratio $\ln(p\left(q_{E}^{R}\right)/p\left(-q_{E}^{R}\right))/\tau$ evaluated at different sampling times τ up to $\tau =10^{3}$ in the main figure and between $\tau =10^{4}$ and $\tau =3\times 10^{4}$ in the inset, as denoted by the legend. The main plot and inset were obtained using data from the main plot and inset of panel (**b**), respectively, and both report the trend of $\left(T_{1}^{-1}-T_{FT}^{-1}\right)\cdot q_{E}$ as a black solid line, with $T_{FT}∼0.3$ extracted from a fit of the curves in the inset performed as described in the main text. (**d**): Overview of the temperatures $T_{FT}^{q_{E}^{R}}$ and $T_{FT}^{q_{W}^{R}}$ (yellow and green circles) extracted from $p(q_{E}^{R})$ and $p(q_{W}^{R})$ at sampling time $\tau =3\times 10^{4}$ as prescribed by Equation (21) as a function of Pe compared to $T_{kin}^{h},T_{eff}^{h}$ and $T_{kin}^{dw},T_{eff}^{dw}$ obtained in the harmonic (light blue and red lines) and double-well (dark blue and red lines) configurations, respectively. $T_{kin}^{h}$ and $T_{eff}^{h}$ are plotted as solid lines to highlight their analytical origin from Equations (A12) and (A13), while all other data are plotted as dot and lines, the dots reporting the values obtained numerically, the lines being a guide to the eye, including the lower black solid line reporting a sample linear trend ∼Pe. In all panels we fixed γ=10 and $T_{1}=T_{2}=0.2$, while in the harmonic and double-well configurations, we set k=4.0 and a=1.0,b=2.0, respectively.

In this section, we investigate case (b) envisaging a left bath which is given an active character through the introduction of an additional Ornstein–Uhlenbeck noise. In Figure 6a, we preliminarily show the distribution $p(q_{E}^{R})$ for $T_{1}=T_{2}=0.2$ and three different Pe at sampling time $\tau =3\times 10^{4}$ (the distributions $p(q_{E}^{L})$ are just symmetrical). The figure is clearly reminiscent of Figure 4a, with Pe effectively playing the role of a temperature like $T_{2}$: as Pe is increased, the distributions shift towards the right, with a consequent increase in their skewness as well as of the average value of $q_{E}^{R}$.

Let us consider in detail the case Pe=50.0. As in Section 3.1, for the right well, the residence time is $\tau_{r}^{r}∼1.64\times 10^{3}$ as prescribed by Equation (11). For the left well we instead numerically estimate it as $\tau_{r}^{l}∼34.18$, so that the conditions $\tau_{r}^{l},\tau_{r}^{r}>\tau_{p}=16.67\gg \tau_{I}=0.1$ are satisfied. Figure 6b shows the trend of $-\ln(p\left(q_{E}^{R}\right))/\tau$ for an increasing sampling time τ, as denoted by the legend. As remarked by the inset and similarly to Section 3.1, also in this case, we find the curves to converge at large times towards a convex rate function $I(q_{E}^{R})$, thus proving $q_{E}^{R}$ satisfies a large-deviation principle even when one of the baths is made active. Figure 6c shows instead the ratio $\ln(p\left(q_{E}^{R}\right)/p\left(-q_{E}^{R}\right))/\tau$ evaluated at different times using data from Figure 6b. Interestingly, also in this case, the resulting curves show a linear trend at all times. Here, the effect of the sub-exponential contribution $c(q_{E}^{R})$ makes the slope of the curves reduce until reaching a constant value, as remarked by the inset. Following the same line of action as in Section 3.1, one can fit these lines as prescribed by Equation (21), so as to extract a temperature estimate for the right well based on fluctuation theorems. When doing so, identifying a priori $T_{r}$ with $T_{1}=0.2$, one finds for the left well $T_{FT}^{q_{E}^{R}}∼0.3$ (whence the choice of parameters in Figure 3b for which $T_{2}=0.3$ from case (a) and $T_{FT}^{q_{E}^{R}}∼0.3$ from case (b) essentially coincide). The resulting line $\left(T_{1}^{-1}-{\left(T_{FT}^{q_{E}^{R}}\right)}^{-1}\right)\cdot q_{E}^{R}$ is reported in Figure 6c for completeness. The possibility $T_{l}=T_{2}=0.2$ can thus be trivially discarded since, as in the case Pe=5 from Figure 6a in which the effect of the active noise is essentially negligible, it would lead to $p\left(q_{E}\right)=p\left(-q_{E}\right)$, and therefore to a vanishing slope.

At this point, one could naturally ask how $T_{FT}^{q_{E}^{R}}$ is influenced by the strength of the activity, i.e. by Pe, and also whether this temperature coincides with other out-of-equilibrium temperatures like the kinetic and effective ones mentioned in Section 1 and detailed in Appendix A. Concerning the first question, Figure 6d shows the trend of $T_{FT}^{q_{E}^{R}}$ (yellow dots) obtained by varying Pe while keeping fixed all other system parameters, while Table 1 reports the exact values emerging from the analytical expressions from Appendix A and from our fit procedure performed while keeping $T_{r}=T_{1}=0.2$ fixed. The data suggest that $T_{FT}^{q_{E}^{R}}$ increases roughly linearly as a function of Pe. Note that at all Pe considered, the estimated left residence times $\tau_{r}^{l}$ satisfy the condition $\tau_{r}^{l}>\tau_{p}=16.67\gg \tau_{I}=0.1$, as reported in Table 1.

In order to answer the second question, we instead compared $T_{FT}^{q_{E}^{R}}$ with the kinetic and effective temperatures obtained for a particle subjected everywhere to the active bath and under the separate action of two different external potentials. The first potential we considered was a harmonic one $U\left(x\right)=kx^{2}/2$ introduced in such a way as to approximate the quartic double-well potential Equation (2) around one if its minima, i.e. setting k=2b with 2b the second derivative of Equation (2) at its minima locations $\pm x_{m}$. In such a case, we referred to the overall configuration as the harmonic configuration, the two temperatures were denoted as $T_{eff}^{h}$ and $T_{kin}^{h}$, and, as detailed in Appendix A, an analytical derivation was feasible with resulting expressions provided by Equations (A12) and (A13). The second potential we considered was instead the usual quartic double-well potential Equation (2), and the relative configuration was referred to as the double-well configuration. Here, the two temperatures were denoted as $T_{eff}^{dw}$ and $T_{kin}^{dw}$ and their estimates were obtained by numerical means. The rationale underlying these configurations followed our desire to understand if the value of $T_{FT}^{q_{E}^{R}}$ was determined mostly by the permanence of the particle around the potential minimum (hence the harmonic configuration), or, similarly to what was observed for the variation in entropy production for an active particle under the action of a quartic double-well potential [70,89], by the non-convex region of Equation (2) (hence, the double-well configuration).

Figure 6d reports the trend of the temperature values we obtained in the two configurations under consideration, while Table 1 offers an overview of their values. From a comparison with the trends and values of $T_{FT}^{q_{E}^{R}}$ it is immediate to realise that there is no correspondence between any of the two effective temperatures. The kinetic temperatures are instead much closer to $T_{FT}^{q_{E}^{R}}$, with $T_{kin}^{dw}$ essentially coinciding exactly. We therefore conclude that it is not enough to limit our attention to the evolution of the particle around the potential minima, but rather considering its dynamics around its local maximum at $x_{u}$ is essential. Moreover, the affinity of $T_{FT}^{q_{E}^{R}}$ to the kinetic temperature seems to mirror the inherent character of $Q_{E}^{R}$: instantaneous energy exchanges are best described in terms of an instantaneous out-of-equilibrium temperature.

**Figure 7:**
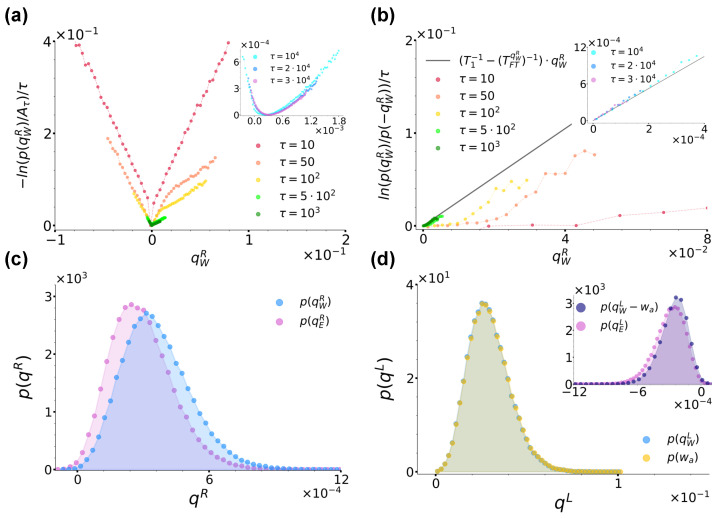
(**a**): Curves $-\ln(p\left(q_{W}^{R}\right))/\tau$ for Pe=50 at different sampling times, as denoted by the legend. $A_{\tau }$ denotes the maximum of the distribution at each sampling time. The inset shows instead the trend of the same curves at the largest sampling times considered. (**b**): Ratio $\ln(p\left(q_{W}^{R}\right)/p\left(-q_{W}^{R}\right))/\tau$ evaluated at different sampling times using data from panel (**a**) along with the right hand-side of Equation (21) with $T_{r}=0.2$ fixed and $T_{l}=T_{FT}^{q_{W}^{R}}∼0.42$ extracted from a fit of the curves in the inset performed as described in the main text, as denoted by the legend. (**c**,**d**): Comparison between the distributions $p\left(q_{E}^{R}\right),\;p\left(q_{W}^{R}\right)$ and $p\left(q_{W}^{L}\right),\;p\left(w_{a}\right)$ at sampling time $\tau =3\times 10^{4}$, respectively, with the same parameters as in Figure 6a. In panel (**d**), the inset shows instead a comparison between $p(q_{E}^{L})$ and $p(q_{W}^{L}-w_{a})$. In all panels, we fixed γ=10, $T_{1}=T_{2}=0.2$ and a=1.0,b=2.0.

We now turn to comment on the behaviour of the heat $q_{W}$ and active work $w_{a}$ per unit time. Figure 7a reports the curves $-\ln(q_{W}^{R})/\tau$ for an increasing sampling time τ for the same parameter choice as in Figure 6b. Along with its inset, the figure shows that also in this case, these curves converge at large times towards a convex rate function $I(q_{W}^{R})$, thus proving that $q_{W}^{R}$ also satisfies a large-deviation principle when the left bath is active. Concerning the validity of a fluctuation theorem, Figure 7b shows the ratio $\ln(p\left(q_{W}^{R}\right)/p\left(-q_{W}^{R}\right))/\tau$ evaluated at different times using data from Figure 7a. As in Figure 5d, at small times, the sub-exponential contribution $(c\left(q_{W}^{R}\right)-c\left(-q_{W}^{R}\right))/\tau$ makes the resulting curves actually curvilinear, while at large times, they assume a linear trend with a constant slope. Following the usual fitting procedure, in this case, we found $T_{FT}^{q_{W}^{R}}∼0.42$, and the resulting curve $\left(T_{1}^{-1}-{\left(T_{FT}^{q_{W}^{R}}\right)}^{-1}\right)\cdot q_{W}^{R}$ is reported in Figure 7b and its inset for completeness. In order to give context to this finding, Figure 7c reports a comparison between $p(q_{W}^{R})$ and $p(q_{E}^{R})$ at sampling time $\tau =3\times 10^{4}$. Interestingly, contrary to Figure 5b from case (a), here, at large times, the two distributions do not coincide, but $p(q_{W}^{R})$ is rather slightly shifted towards the right with respect to $p(q_{E}^{R})$. As Figure 6b and Figure 7a show that at this τ, the curves $-\ln(p\left(q_{E}^{R}\right))/\tau$ and $-\ln(p\left(q_{W}^{R}\right))/\tau$ have both already converged towards their respective rate functions, the origin of this discrepancy is not a matter of not a long enough sampling time, but its explanation must rather be searched once again in the very dynamics of the system. To this end, we reconsidered the position stationary distribution from Figure 3b. As commented in Section 2.4, this is characterised by a left peak shifted towards the left with respect to the location of the left minimum $-x_{m}$ of the double-well potential Equation (2). This effect is in turn ascribable to the action of the active noise a(t) which pushes the particle towards the left when assuming persistently negative values. When instead a(t) persistently assumes positive values, it pushes the particle towards the right until making it jump in the right well. In doing so, the particle essentially takes a run-up, so that, contrary to case (a), when jumping towards the right, its velocity is enhanced. As a consequence, the particle is not able to dissipate all accumulated excess energy essentially instantaneously at $x_{u}$ as in case (a), but it rather completes its thermalization with the right-well bath during the descent towards the right minimum located at $x_{m}$, hence the released surplus energy, the shift of $p(q_{W}^{R})$ and the resulting fit temperature $T_{FT}^{q_{W}^{R}}$ higher than both $T_{FT}^{q_{E}^{R}},T_{kin}^{dw}∼0.3$.

We remark that this finding is coherent with the phenomenology described above: the release of energy associated with $q_{W}^{R}$ does not occur instantaneously, so the kinetic temperature is obviously not fit to describe this phenomenon. At the same time, this energy release does not persist long enough to make the effective temperature $T_{eff}^{dw}∼21.2$ intervene, so that $T_{kin}^{dw}∼T_{FT}^{q_{E}^{R}}<T_{FT}^{q_{W}^{R}}<T_{eff}^{dw}$. Interestingly, Figure 6d and Table 1 show that the temperature discrepancies we uncovered at Pe=50 are not peculiar of this specific case but are instead common for all other Pe we considered, with $T_{kin}^{dw}∼T_{FT}^{q_{E}^{R}}<T_{FT}^{q_{W}^{R}}<T_{eff}^{dw}$ in all cases.

To conclude, we briefly comment on the heat exchanges’ distributions for the left bath, captured at sampling time $\tau =3\times 10^{4}$ and Pe=50. Figure 7d reports $p(q_{W}^{L})$ and $p(w_{a})$ and shows that values spanned by these distributions are much larger than the ones spanned by $p(q_{W}^{R})$ and $p(q_{E}^{R})$ from Figure 7c. This effect is due to the enhanced velocity of the particle pushed by the active force. Note also that the signs of $q_{W}^{L}$ and $w_{a}$ agree and their distributions almost overlap. That they do not completely overlap is in turn shown by the inset reporting $p(q_{W}^{L}-w_{a})$ compared to $p\left(q_{E}^{L}\right)=p\left(-q_{E}^{R}\right)$. The inset in fact shows that $p(q_{W}^{L}-w_{a})$ records non-zero values of three orders of magnitude lower than those associated with the distributions from the main figure and of the same order of magnitude as the ones associated with $p(q_{E}^{L})$. Moreover, similarly to what happens for $p(q_{W}^{R})$ and $p(q_{E}^{R})$ from Figure 7c, $p(q_{W}^{L}-w_{a})$ is slightly shifted with respect to $p(q_{E}^{L})$. Finally, we conclude by remarking that the distributions $p(q_{W}^{R})$ and $p(q_{W}^{L}-w_{a})$ are coherent with the energy balance Equation (19).

## 4. Conclusions

In this paper we numerically studied the heat exchanges occurring between two heat baths of different nature with the purpose to investigate the role temperature plays in these phenomena. The baths were spatially confined in the two wells of a quartic double-well potential, while the heat exchanges were mediated by a Brownian particle jumping between the two. Heat was sampled according to two different definitions: as the total kinetic energy carried by the particle when jump events occur and as the work performed by the particle on one of the two baths when immersed in it. These heat distributions were used to check the validity of a fluctuation theorem whence possibly extracting a temperature estimate for the baths through a proper linear fit. This procedure allowed us to introduce the definition of an out-of-equilibrium temperature whose resulting values we compared not only with the bath temperatures, but also with other out-of-equilibrium temperatures as the kinetic and effective ones. Operatively, we fixed an equilibrium bath in the right well and considered two different configurations for the one in the left well.

In the first case, we fixed another equilibrium thermal bath with a different temperature and found both heat definitions to satisfy the same fluctuation theorem with fit temperatures coinciding with the ones of both baths. These results extend the analysis of [53,57,58] to the cases of spatially separated equilibrium thermal baths.

In the second case, we instead considered an active bath by introducing an additional Ornstein–Uhlenbeck noise, making the bath effectively out of equilibrium. Also in that case, we found a fluctuation theorem to be satisfied. However, there, the temperature relative to the left bath turned out to coincide with its out-of-equilibrium kinetic temperature for the heat defined as the sum of kinetic energies and with a higher one yet still lower than the effective temperature for the other definition of heat. These results and discrepancies were interpreted by looking at the system phenomenology, finding them to mirror the instantaneous or longer release of energy captured by both heat definitions.

The present study could represent the first step towards a deeper and wider investigation on the role played by temperature in heat exchanges. If and where possible, analytical approaches could in fact provide further validation and insights into the overall scenario emerging from our investigation. Moreover, more complex geometries and bath features could be explored so as to clarify even better the role of kinetic temperature in heat exchanges or reveal cases where instead the effective temperature plays a prominent role. Experiments adopting setups and technologies already in place like Janus particles [72,73,74,75] and optical tweezers [76,77] could provide further validation and connections with real systems.

## Figures and Tables

**Table 1:** Estimates of the average residence time in the left well $\tau_{r}^{l}$ and of the temperatures $T_{q_{E}^{R}}$, $T_{q_{W}^{R}}$, $T_{eff}^{h}$, $T_{kin}^{h}$, $T_{eff}^{dw}$, $T_{kin}^{dw}$ for various choices of increasing Pe. The values of $T_{eff}^{h}$, $T_{kin}^{h}$ were obtained analytically from Equations (A12) and (A13), while all other time and temperature estimates were obtained numerically. In all cases, the system was evolved until $\tau =3\times 10^{4}$, and we fixed γ=10 and $T_{1}=0.2$, $T_{2}=0.2$, while in the harmonic and double-well configurations, we set k=4.0 and a=1.0,b=2.0, respectively.

Pe	$\tau_{r}^{l}$	$T_{\mathrm{FT}}^{q_{E}^{R}}$	$T_{\mathrm{FT}}^{q_{W}^{R}}$	$T_{\mathrm{eff}}^{h}$	$T_{\mathrm{kin}}^{h}$	$T_{\mathrm{eff}}^{\mathrm{dw}}$	$T_{\mathrm{kin}}^{\mathrm{dw}}$
50.0	34.18	0.30	0.42	22.05	0.33	21.20	0.30
65.0	32.09	0.35	0.45	37.13	0.42	30.68	0.35
75.0	31.41	0.39	0.47	49.36	0.49	32.92	0.38
100.0	29.85	0.43	0.60	92.42	0.72	37.49	0.46
150.0	27.36	0.63	1.59	207.69	1.43	97.12	0.66

## Data Availability

Data can be made available upon reasonable request.

## Appendix A. Effective and Kinetic Temperatures

*Effective* and *kinetic* temperatures are two out-of-equilibrium temperature definitions able to capture instantaneous and time-delayed properties of the system [3,4,40,41,45,46,59,60,61,62], respectively. Their definitions rely in fact on two fundamental results of statistical mechanics more effective in these two time regimes: the equipartition theorem [90] and the fluctuation–dissipation theorem [91].

Concerning the effective temperature, we first recall the definition of mean square displacement and integrated linear response function. The former is defined as

(A1) $$\Delta^{2}\left(t^{\prime },t\right)\equiv \langle {\left[r\left(t\right)-r\left(t^{\prime }\right)\right]}^{2}\rangle \;,$$

and measures how far on average a particle travels over time with respect to a fixed initial location, while the latter is defined as

(A2) $$\chi \left(t^{\prime },t\right)\equiv \int_{t^{\prime }}^{t}dt^{\prime \prime }\sum_{\alpha =1}^{d}R_{\alpha \alpha }\left(t^{\prime \prime },t\right)\;,$$

with

(A3) $$R_{\alpha \beta }\left(t^{\prime },t\right)=\frac{\delta {\langle r_{\alpha }\left(t\right)\rangle }_{h}^{\lambda }}{\delta h_{\beta }^{\lambda }\left(t^{\prime }\right)}{|}_{h_{\beta }^{\lambda }=0}$$

the linear response of the system, *d* the dimension of the system, α,β dimensional indices and $h_{\beta }^{\lambda }\left(t^{\prime }\right)$ an external perturbation depending on the parameter λ and measures how a system responds to a small external perturbation. The above two functions are both involved in the position fluctuation–dissipation theorem

(A4) $$2T\chi \left(t^{\prime },t\right)=\Delta^{2}\left(t^{\prime },t\right)\;.$$

Based on the above relation, the time-dependent effective temperature for an out-of-equilibrium system is then defined as [3,4,61]

(A5) $$T_{eff}\left(t^{\prime },t\right)\equiv \frac{\Delta^{2}\left(t^{\prime },t\right)}{2\chi (t^{\prime },t)}\;.$$

Concerning the kinetic temperature, we recall the equipartition theorem stating that for each degree of freedom *i* of an equilibrium system, the temperature *T* and the velocity fluctuations are related as

(A6) $$\frac{1}{2}m\langle {\dot{r}}_{i}^{2}\left(t\right)\rangle =\frac{1}{2}k_{B}T\;.$$

It is then natural for each degree of freedom of an out-of-equilibrium system to define the time-dependent kinetic temperature as [4,46,62]

(A7) $$T_{kin}\left(t\right)\equiv \frac{m\langle {\dot{r}}_{i}^{2}\left(t\right)\rangle }{k_{B}}\;.$$

In equilibrium systems, as for example a single Brownian particle in contact with a white-noise bath with temperature *T*, the two definitions boil down to the same expression, *T*. In more complex configurations, they can instead be very different. For example, for a free active Ornstein–Uhlenbeck particle [63,64,65,66], i.e., a particular instance of an active particle, the integrated linear response function reads

(A8) $$\chi \left(t^{\prime },t\right)=\frac{t-t^{\prime }}{\gamma }\;,$$

while the mean square displacement and velocity expressions are provided by [64]. Combining these functions as prescribed by Equations (A5) and (A7), and fixing $t^{\prime }=0$, one finds that in the long time limit t↑∞

(A9) $$T_{eff}\;⟶\;T+\frac{F_{a}^{2}}{\gamma \gamma_{R}}\;.$$

and

(A10) $$T_{kin}\;⟶\;T+\frac{F_{a}^{2}}{\gamma }\frac{1}{\left(\gamma_{R}+\frac{\gamma }{m}\right)}\;,$$

where we dropped the time dependence as both temperatures reach a constant value. Analytical estimates can also be provided for an active Ornstein–Uhlenbeck particle under the action of an external harmonic potential like $U\left(x\right)=kx^{2}/2$. In this case the integrated linear response function is

(A11) $$\chi \left(t^{\prime },t\right)=\frac{1-e^{-\frac{\gamma }{k}\left(t-t^{\prime }\right)}}{k}\;,$$

while the mean and velocity square displacement expressions are again provided by [64]. Combining these functions as above, and fixing $t^{\prime }=0$, one finds that in the long time limit t↑∞

(A12) $$T_{eff}\;⟶\;T+\frac{F_{a}^{2}}{\gamma }\frac{1+\frac{\gamma }{m\gamma_{R}}}{\left(\gamma_{R}+\frac{\gamma }{m}+\frac{k}{m\gamma_{R}}\right)}\;.$$

and

(A13) $$T_{kin}\;⟶\;T+\frac{F_{a}^{2}}{\gamma }\frac{1}{\left(\gamma_{R}+\frac{\gamma }{m}+\frac{k}{m\gamma_{R}}\right)}$$

which in the limit k↓0 reduce to Equations (A9) and (A10), respectively.

To our knowledge, in even more complex systems analytical results are not available. However, one can always resort to numerical methods and evaluate the kinetic and effective temperatures as prescribed by their definitions Equations (A5) and (A7). In particular, the effective temperature estimation requires the knowledge of the integrated linear response function to be evaluated using an external perturbation low enough for the system to remain in the linear regime and at the same time large enough to overcome large fluctuation effects.
